# Ferroelectric domain wall motion induced by polarized light

**DOI:** 10.1038/ncomms7594

**Published:** 2015-03-17

**Authors:** Fernando Rubio-Marcos, Adolfo Del Campo, Pascal Marchet, Jose F. Fernández

**Affiliations:** 1 Electroceramic Department, Instituto de Cerámica y Vidrio, CSIC, Kelsen 5, Madrid 28049, Spain; 2 Laboratoire de Science des Procédés Céramiques et de Traitements de Surface, UMR 7315 CNRS, Université de Limoges, Centre Européen de la Céramique, 12, rue Atlantis, Limoges 87068, France

## Abstract

Ferroelectric materials exhibit spontaneous and stable polarization, which can usually be reoriented by an applied external electric field. The electrically switchable nature of this polarization is at the core of various ferroelectric devices. The motion of the associated domain walls provides the basis for ferroelectric memory, in which the storage of data bits is achieved by driving domain walls that separate regions with different polarization directions. Here we show the surprising ability to move ferroelectric domain walls of a BaTiO_3_ single crystal by varying the polarization angle of a coherent light source. This unexpected coupling between polarized light and ferroelectric polarization modifies the stress induced in the BaTiO_3_ at the domain wall, which is observed using *in situ* confocal Raman spectroscopy. This effect potentially leads to the non-contact remote control of ferroelectric domain walls by light.

## Abstract (web summary)

Domain walls between ferroelectric domains are of interest for ferroelectric memory and to achieve a better control of the switching process. Here, the authors induce the motion of ferroelectric domains by light, creating a new possibility to control ferroelectrics.

## Graphical abstract

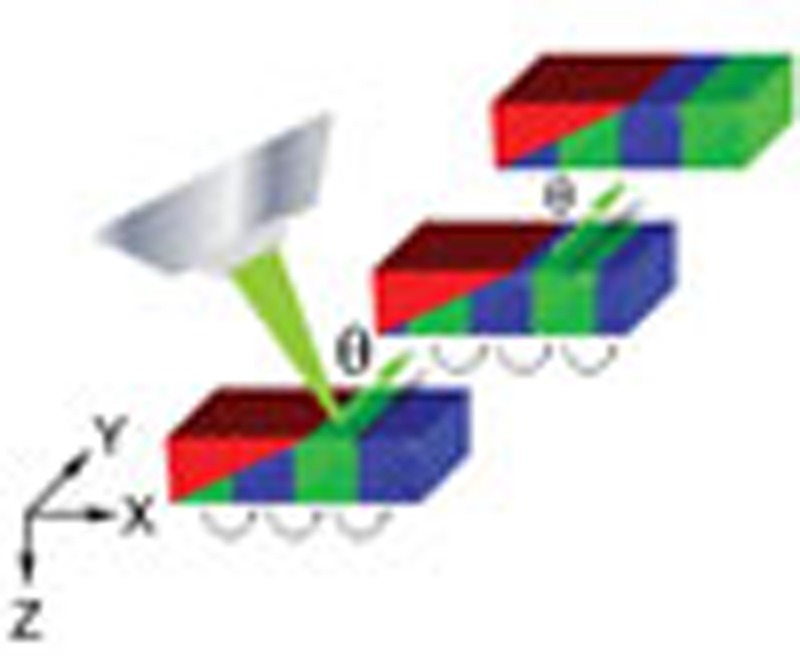

Ferroelectric devices, as nonvolatile ferroelectric random access memory (FeRAM)^1^, are based on the electrically switchable nature of the spontaneous polarization, which is characteristic of the ferroelectric materials. In such memory devices, the storage of data bits is achieved by driving domain walls that separate regions with different polarization directions. Thus, an external voltage pulse can switch the polarization between two stable directions, representing ‘0’ and ‘1’. This simple behaviour is responsible for the read/write process that can be completed within nanoseconds. The major drawback of FeRAM is that they require circuitry access. Unfortunately, practical implementations of FeRAM for commercial use are still limited by difficult integration into devices as compared with their conventional magnetic RAM counterparts^2^.

For this reason, the ability of moving domain walls in ferroelectric materials without a physical contact fascinates the scientific community. This motivation has induced an important research activity in multiferroic materials, combining the advantages of both ferroelectricity and ferromagnetism for new nonvolatile memories, which can be written electrically, but read magnetically. Recently significant room temperature coupling was demonstrated by monitoring changes in ferroelectric domain patterns induced by magnetic fields^3^. The room temperature multiferroicity and magneto-electric coupling was demonstrated unambiguously in epitaxial thin films of BiFeO_3_ (ferroelectric polarization ~100 μC cm^−2^ and saturation magnetization of 150 emu cm^−3^)^4^. One of the drawbacks of multiferroic oxides is the presence of electronic conductivity in ferroelectric domain walls^5^,^6^,^7^,^8^. However, the effect is attractive for photovoltaic devices^9^ because large photovoltages can be generated by domain walls. The photovoltaic effect has been reported in ferroelectric materials some decades ago^10^ and there is renewed interest due the renewable solar energy use.

Recently, in a pioneering study, Sluka*et al*.^11^ demonstrated the existence of ‘strongly’ charged domain walls (sCDW) in the ubiquitous ferroelectric BaTiO_3_ (BTO) showing electron gas-like conductivity, while the individual domains remained excellent insulators. The discovery of this stimulant behaviour from the sCDW described for the BTO-based material^11^ and the potential technological applications from these enhanced functionalities, raise a fundamental question: How can these functional sCDW be tuned with a large degree of control in the mobility of the domain walls? In fact, charged domain walls are expected to be ‘heavy’ and to be less effectively influenced by pressure^12^,^13^. Thus far, it has been observed that ferroelectric but simultaneously ferroelastic charged domain walls are strongly pinned in real space in BiFeO_3_ thin films^14^, which are perovskite-type multiaxial ferroelectric.

The interaction of light with matter above described for the photovoltaic effect can be further exploited by the generation of photostriction on ferroelectric materials. The photostrictive effect is a phenomenon in which strain is induced in the sample when illuminated^15^,^16^,^17^. Thus, photostriction in ferroelectrics materials arises from a superposition of photovoltaic and inverse piezoelectric effects^15^,^16^,^17^. However, since 1970, s, BTO single crystals have been given great attention owing to their extrinsic photoconductivity, which is conditioned by the effect of a strong electric field in the surface layer of the quantum yield and the lifetime of non-equilibrium carriers.^18^,^19^

Here we have made a breakthrough that could lead to devices without electrical connections, directly converting energy from light into ferroelectric domain wall motion. To achieve this purpose, we have correlated an unexpected coupling between polarized light and ferroelectric polarization, which modifies the stress induced at the domain wall. As a result, these properties are particularly attractive, because domain walls move without applying an electrical field and the process is reversible accordingly with the intrinsic ferroic nature of the crystal. This new effect potentially leads to remote control of ferroelectric domain walls by light, which opens a framework for micro and nanoscale devices with non-contact domain engineering.

## Results

### Characterization of the domain structure by AFM

**Figure 1:**
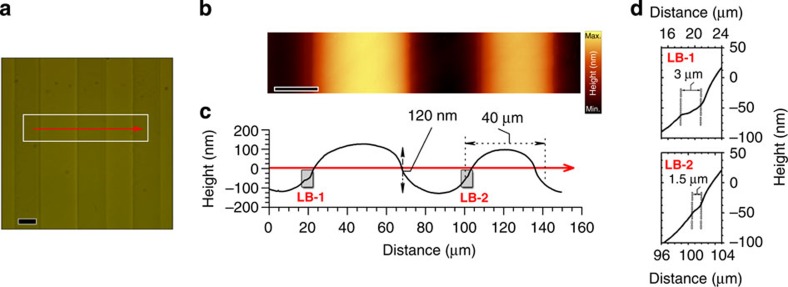
Characterization of the BTO single crystal by optical microscopy and AFM (**a**) optical micrograph of the domain structure. (**b**) AFM image of BTO crystal, showing the domain topography inside the marked white box of **a**. Scale bar, 20 μm. (**c**) AFM topography scan along the red arrow of **a**. (**d**) Detail of the domain boundary topography, corresponding to the left boundaries **LB-1** and **LB-2** of **b**.

A basic identification of the structure and of the crystalline orientation of the single crystal was performed using X-Ray Diffraction (see Supplementary Fig. 1 and Supplementary Table 1). The sample presents a tetragonal symmetry and two different crystallographic orientations, (00 l) or *c*-plane and (h00) or *a*-plane. Study of the domain structure was performed by optical microscopy and atomic force microscope (AFM). Figure 1a depicts an optical microscopy image of the BTO sample aligned perpendicularly to the AFM cantilevers. The area of 150 × 30 μm (Fig. 1a) delimits the range where topographic information was collected by AFM. Figure 1b shows a detailed AFM topographic image of the domain structure. The AFM scan along the red arrow of Fig. 1a is illustrated in Fig. 1c. The domain structure is mainly composed of domains of 400 to 50-μm width and adjacent domains appear alternately as protrusions and troughs (height difference of ~120 nm). The AFM scan also reveals the domain boundary topography, associated with soft transitions (Fig. 1d). Moreover, such transitions are asymmetric: left boundary of the protrusions presents a characteristic step (1.5–3 μm), while this step is not observed for right boundary.

### Mapping of domains by Confocal Raman Microscopy

**Figure 2:**
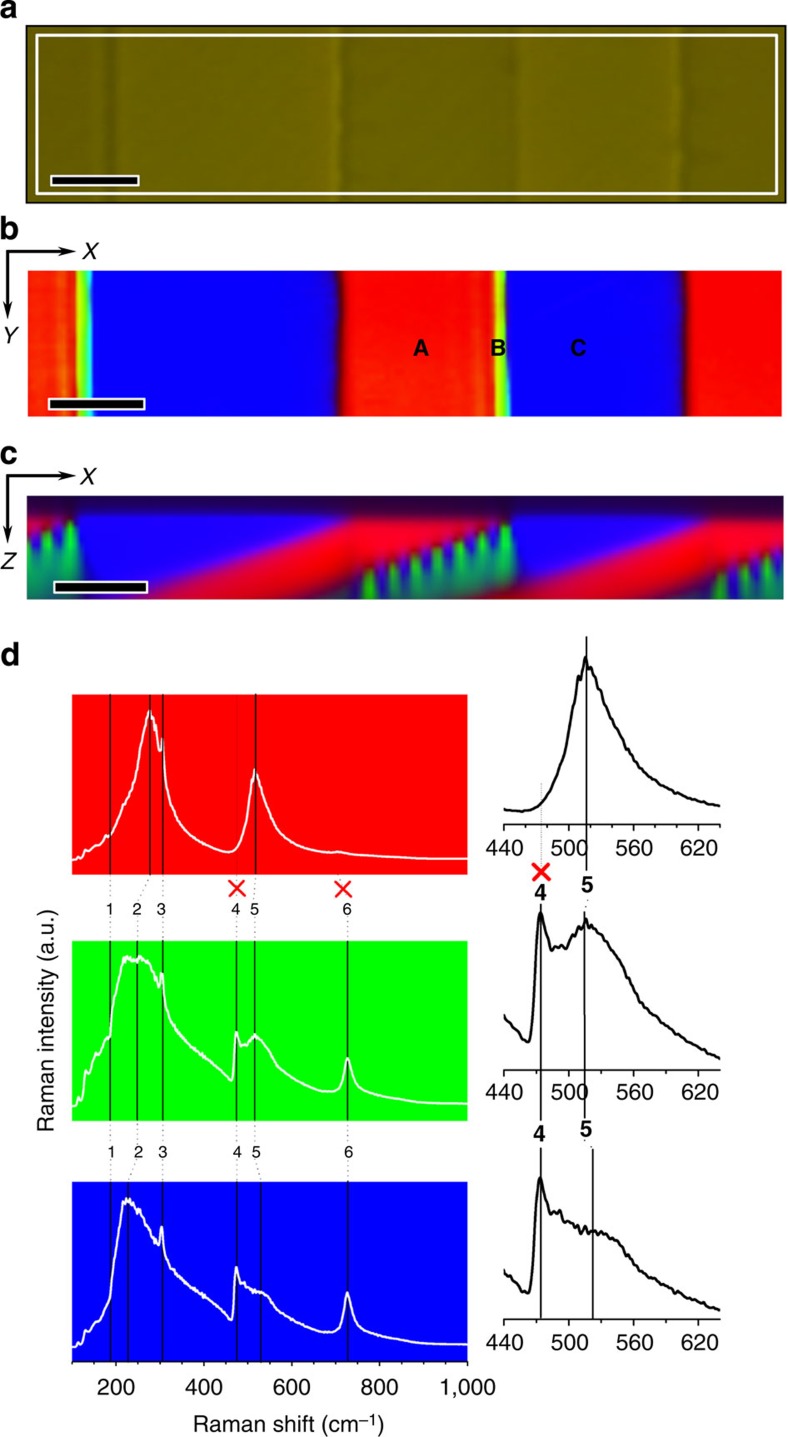
Mapping of the domain structure of the BTO single crystal through confocal Raman microscopy: (**a**) optical micrograph of the BTO single crystal. The white rectangle of Fig. 1a shows the positions where the *XY* Raman image and *XZ* Raman depth scan image are performed and correspond with the area of previous AFM analysis. The Raman image shows the domain distribution at the surface by colour code (**b**) as well as in the depth scan, cross section, (**c**). The Raman images resulted from the mapping of the different single Raman spectra collected in each pixel of the marked rectangle area in **a**. Raman spectra having same spectral shift for the Raman modes are identified using the same colour. The intensity of the colour is correlated with the Raman intensity. Scale bar, 20 μm. (**d**) Main Raman spectra of BTO Raman image associated with the three different colours: red=*a*-domain, blue=*c*-domain, green=*b*-domain, which are collected in the points marked as **A**, **C** and **B** in (**b**) respectively. The numbers next to the vibrational peaks represent the main atomic motions (for the assignment of the Raman modes see Supplementary Table 2). The inserts show magnified Raman spectra, ascribed to the **4** and **5** Raman modes, respectively.

The AFM technique gives topographical information of the domain structure but yields no structural information. Recent methods based on confocal Raman microscopy (CRM), give the possibility to study at a local scale the structural deformations of perovskites, induced both by the tilting of BO_6_ octahedra and by the cationic displacements^20^. CRM is here combined with AFM in the same apparatus, thus giving direct correlations between topography and local structure. Figure 2a depicts an optical micrograph of the polished surface of the crystal, aligned perpendicularly to the Raman laser. The Raman spectra are collected at a plane located just below the surface of the sample, where the Raman intensity is maximized. The selected area (150 × 30 μm) is the one previously studied by AFM. The acquisition time for a single Raman spectrum was 1 s (1 pixel). Thus the Raman image consisting of 150 × 30 pixels (4,500 spectra) required 75 min for the planar section. Features such as Raman peak intensity, peak width or Raman shifts are fitted with algorithms to compare information and to represent the derived Raman image. The assignments of the observed Raman modes, both symmetry and nature (first and second order), are summarized in the Supplementary Table 2. Raman spectra having same Raman shift are classified by colours and the colour intensity corresponds to the Raman intensity. The combination of colours results in a Raman image of the surface (Fig. 2b) and a Raman depth scan image of the cross-section (Fig. 2c). Figure 2d shows the average Raman spectra obtained in adjacent domains: **A** (red) and **C** (blue) points.

### Crystalline orientation of red and blue domains

Clear differences can be observed in the shape of both spectra, particularly in the 440 to 640 cm^−1^ region (see details for bands **4** and **5** in Fig. 2d), and ~720 cm^−1^ (Raman mode signalled as **6** in Fig. 2d). The *a*-domains are easily detectable since they are characterized by the annihilation of two Raman modes (**4** and **6** in Fig. 2d). In addition, Pezzotti *et al*.^21^ have recently reported a strong asymmetry of the A_1_(TO_3_) band (**5** in Fig. 2d) ~520 cm^−1^. This asymmetry is characteristic of the *a-plane* of BTO tetragonal phase^21^ and required a deconvolution into a sharp and a broad band (510 cm^−1^ and 560 cm^−1^, respectively). Raman peaks at 520 and 560 cm^−1^ are attributed to the same A_1_(TO_3_) Raman mode, even if the physical nature of band asymmetry is not yet established. Consequently, the red spectrum of the Fig. 2d allows the assignment exclusively to *a*-domain. Thus, red and blue spectra correspond, respectively, to *a*- and *c*-domains.

In addition, there is a clear correlation between *c*-domain regions (out-of-plane polarizations) and the protrusion evidenced by AFM, as well as between *a*-domain areas (in-plane polarization) with the trough determined by AFM. Generally, the plane of *a–c*-domain walls is {110}_pc_ at 45° to the top surface^22^,^23^. This phenomenon results from twinning structurally induced by the ferroelastic–ferroelectric phase transition occurring at 120 °C in BTO (high temperature *Pm3m* cubic→tetragonal *P4mm*). The 90° ferroelastic domains are generated, each of them gives rise to two 180° ferroelectric domains. As a consequence, domain boundaries are along {110}_Cubic_ lattice planes, with ≈45° angle between the boundary and the (100)_Tetragonal_ *a*-plane or the (001)_Tetragonal_ *c*-plane of the tetragonal cell^23^. Thus the alternate *a–c*-subdomains observed for the single crystal are due to twinning, induced either by synthesis (phase transition) or by the cutting step (ferroelastic transformation)^23^. Here surprisingly, the *a–c*-domain and *c–a*-domain walls slopes ~20° to the top surface. Further experiments are in progress to confirm this result, even if this result seems to be in agreement with recent studies^22^.

### Study of domain boundaries using their Raman spectra

The most unexpected result obtained from the Raman image of the surface is the appearance of a new type of domains (green colour, point **B** in Fig. 2b). This unusual domain boundary can be unambiguously identified by CRM imaging at *a*–*c*-domain wall and is clearly located at the step detected from the topographical analysis (Fig. 1d). For the sake of simplicity, we will use hereafter the term *b*-domain to refer to this boundary. The average width of this *b*-domain is ~1.4 μm, thus larger than the optical resolution of the CRM.

To clarify the complex nature of the *b*-domain, a depth scan Raman image (cross-section), is carried out (Fig. 2c). As a relevant result, we found that *b*-domains are observed only at the *a–c*-domain walls, but never in the *c–a*-domain walls and set of parallel *b*-domains is growing in the *c*-domain region (see the Supplementary Fig. 2 for a detailed configuration of domain boundaries at the cross-section).

The Raman spectrum of the *b*-domain (green colour, point **B** in Fig. 2d) appears as a combination between those of *a*-domains and *c*-domains. The macroscopic symmetry of the sample is tetragonal, but high stress at the domain wall could locally alter symmetry, making Raman-forbidden phonons detectable in local structures^24^.

Unfortunately, characteristic Raman spectra of non-tetragonal BTO phases are not clearly observed. In our case the cutoff filter is 150 cm^−1^, limiting the assignation of low wave number Raman peaks. At low wave number (<200 cm^−1^), there is a slight increase of the background spectra for the *b*-domains, in which incipient Raman peaks at 166 and 199 cm^−1^ seems to appear. The appearance of nanometer-sized local structures with rhombohedral symmetry was recently evidenced in both the orthorhombic and tetragonal phases of BTO single crystal by convergent-beam electron diffraction^25^. Thus, such peaks could be correlated with rhombohedral symmetry (low temperature polymorph)^26^. For rhombohedral phase in modified BTO ceramics, three Raman peaks at 113, 161 and 199 cm^−1^ are attributed to the presence in the lattice of different kinds of BO_6_ octahedra. Nevertheless, other Raman peaks could not be associated with the rhombohedral phase^27^. On the other hand, the monoclinic structure shows a characteristic strong Raman band at 590 cm^−1^, as reported in monoclinic single crystal of BaTi_2_O_5_ (refs 28, 29), which is not observed in our case. To sum up, the crystalline structure of *b*-domain is unequivocally determined as mainly tetragonal, with nanometer-sized local structure presenting high distortion for *b*-domains, observed only in the *c*-domain region at the *a–c*-domain walls. The nanometer-size origin of this distortion could not be unambiguously resolved by the appearance of characteristic Raman peaks.

### Origin of the *b*-domains and model of the domain structure

Taking into account their size, orientation and morphology, the *b*-domains appear different from the domain walls previously observed by optical microscopy in BTO single-crystal^23^. From their Raman signature, the *b*-domains appear clearly as a combination of both the *a*-domain and *c*-domain that could be explained as a bundle of subdomains. Indeed, recent studies have revealed the existence of *a–c*-subdomain structure in thin single-crystalline slices of BTO, referred as ‘superdomains’^30^. The formation of complex domains accounts for the existence of stress in thin layers, that usually relax for thicknesses >100 nm. It is also reported that the piezoelectric response in epitaxial PbZr_(1−x)_Ti_x_O_3_ (PZT) thin films is particularly sensitive to embedded ferroelastic domains and can be dramatically enhanced by manipulating the domain structure to either remove these domains or increase their mobility^31^. Complex domain structures have been also reported for PbTiO_3_ thin films, as predicted by Meyer *et al*.^32^, in addition to a small onset of electrostatic potential across the two sides of a 90° domain wall. Such potential difference (~100 mV) was also evidenced on the surface of a BTO single-crystal at 180° domain wall^33^ and 90° domain wall^34^. The appearance of electrical potential in ferroelectrics should have a dominant effect on the migration of charges that accumulates electron and oxygen vacancies at domain walls. This novel hindering effect has been reported for domain walls in thin films, via the formation of a transient layer with a thickness of several unit cells, as a charged interface between a ferroelastic domain and a switched domain^35^.

The twinned tetragonal model only allows ≈90° and ≈180° angles between polarization vectors of neighbouring domains^23^,^36^. On the contrary, rotation of the polarization between two 90° domains would be allowed by local structures of other symmetries (monoclinic, rhombohedral or orthorhombic)^37^. Such phenomenon was evidenced for PZT ceramics (monoclinic symmetry) at the Morphotropic Phase Boundary^38^. For BTO single-crystal, a rhombohedrally disordered structure was also recently evidenced at the local scale^25^. In our case, the observation of a Raman signature associated to non-tetragonal phase at the domain boundary is thus not surprising. Such *b*-domain would allow the rotation of the polarization. Consequently, this *b*-domain is associated to a non 90° polarization vector and probably electrically charged.

The *b*-domains clearly appear in the *a–c*-domain wall due to higher crystalline stress in the *a–c*-domain wall than in the *c–a* one. Due to the boundary conditions of the crystal surface, the *c*-domain protrudes accordingly with out-of-plane distortion, while the *a*-domain is depleted. The *a*-domain partly overlays the *c*-domain and limits such protrusion. As a result, the *a–c*-domain wall is under compressive stress, minimized by the complex domain structure, which increases the domain density. In the *c–a*-domain wall, the protrusion of the *c*-domain region is favoured because in such case the *c*-domain is located at the surface and the *a*-domain is below it. Thus the *c–a*-domain wall possesses less stress and the complex domain structure is not favoured.

**Figure 3:**
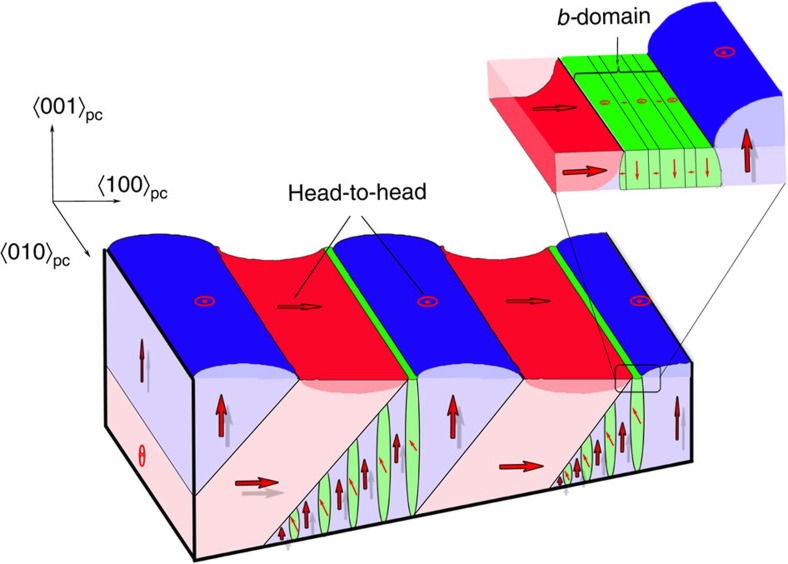
Scheme of the BTO complex domain structure: The Figure displays a schematic illustration of the domain structure, which has been built by combining the AFM and Raman mapping information, shown in Figs 1 and 2, respectively. Scheme shows a domain structure composed of *a*-domain and *c*-domain, which are representend in red and blue colours, with a head-to-head configuration. The head-to-head configuration maximizes the internal stress at close to the domain wall. As a consequence of these internal stresses the *a-c*-domains are hindered by *b*-domains, which are represented in green colour. The insert of the *b*-domains structure shows how internal stress at the domain wall is minimized by a bundle of alternate *a*-domain and *c*-domain.

A phenomenological model describing the formation of the *b*-domain structure is built by combining the AFM and Raman mapping information (Fig. 3). The structure is composed of *a*-domains (red) and *c*-domains (blue). The *b*-domains (green) appear in the *a–c*-domain wall within the ({101}_pc_) plane. The *a–c*-domain wall is associated with a head-to-head 90° domain wall, where the mechanical stress is enhanced. We also envisage that the head-to-head configuration maximizes the bound charge close to the domain wall. The *b*-domains minimize the internal stress by increasing the domain wall density, (insert of Fig. 3). With regards to this discernment, we believe that the *b*-domains are critical to stabilize the *a–c*-domain wall stress and its head-to-head configuration.

Despite the fact that the existence of stress is resolved by increasing domain wall density, *b*-domains appear only in the *c*-domain region and also form domain walls with the *a*-domain. The presence of defects in the 90° domain region (electrons or oxygen vacancies) seems to be behind such behaviour^39^. Indeed, the existence of sCDW^11^ in the BTO single crystal has been recently associated with head-to-head 90° domain wall. The result is the formation of degenerate quasi-two-dimensional electron gas, with metallic free-carrier concentration. Since the band structure of metallic-oxides is extremely sensitive to the surface states, the appearance of the stress promoting the *b*-domain structure must also affect the charge distribution at the domain wall.

### *In situ* domain switching using polarized light source

Following the above analysis, the *b*-domains are associated with a significant stress degree and according to the literature by an uncompensated charge^39^. sCDW generate photovoltages that could imply an interaction of photons with the accumulated charge. Consequently, a response of the sCDW related either to charge or stress redistribution could be expected when exposed to light. To explore such possibility, *in situ* observations of polarization switching are carried out by applying polarized light. In all cases the incidence is normal to the sample surface and the polarization direction of light parallel to the surface. The angle between the polarization of light and the depth scan direction varies by using Δ*θ*=15° between *θ*=0° and *θ*=90°. The scanning direction is always perpendicular to the *a–c*-domain wall. Thus, in the *a*-domain case the polarization of light and thus its associated electric field (*E*) is parallel to in-plane polarization for *θ*=0° (*E* parallel to *P*) and perpendicular for *θ*=90°. Consequently, the electric field have no effect on the polarization vector of BTO cell for *θ*=0° (*E* and *P* are parallel) and acts with a maximum force for *θ*=90° (*E* and *P* are perpendicular). Thus, for the *a*-domain, the effect of the electric field implies in-plane rotation of the polarization vector. The *a*-direction remains parallel to the surface of the sample, which means that this domain remains to be *a*-domain even with re-orientation of its polarization direction under polarized light.

For *c*-domain, the polarization of light is always perpendicular to the out-of-plane polarization. Whatever the *θ* angle, the electric field associated to polarized light have the same effect. For the *b*-domain, a combination of both behaviours is expected as a result of the bundle of *a–c*-subdomains. Thus *b*-domains are probably the most sensitive to electric field and thus to the polarized light.

**Figure 4:**
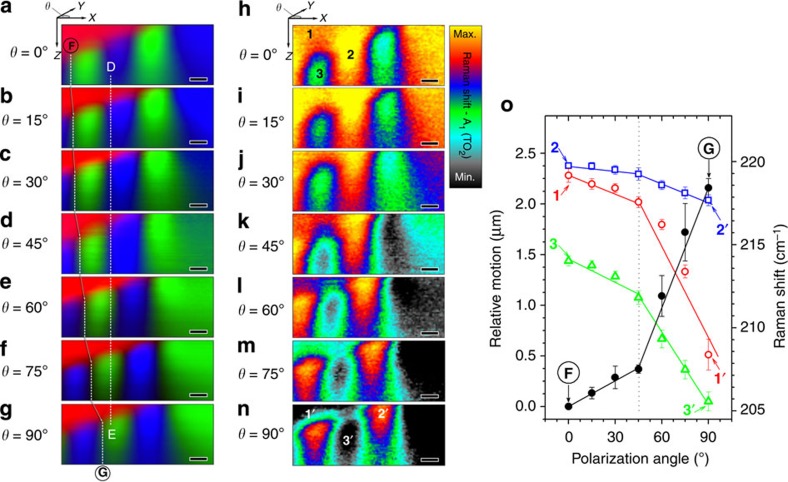
Motion of the ferroelectric domain under polarized light: (**a**–**g**) Sequence of Raman depth scan images showing the switching of the *c*-domain and *b*-domain in the BTO cross-section for different angles of light polarization between 0°**→**90°. The scheme localized at the top of **a** represents the angle *θ* of the polarized light in the plane *XY*. In addition on the left of each image the light polarization angle value is indicated. The Raman spectra taken in two strategic points selected along the line **D**→**E** denoted as **D** and **E** in **a**–**g** are shown in Supplementary Fig. 3. Scale bar, 1 μm. (**h**–**n**) Sequence of Raman shift depth scan images showing the Raman shift at each pixel corresponding to the A_1_(TO_2_) Raman mode **2** in the Supplementary Table 2. The colour code of the bar corresponds to maximum (220 cm^−1^) and minimum (205 cm^−1^) values of Raman shift of **h**–**n**. Scale bar, 1 μm. The relative motion of domain is illustrated along the line marked as **F→G** in **a**–**g**, and is plotted as a function of the polarization angle (*θ*) in the **o**. The error bars show the s.d. of the relative motion from the measurement for each angle of light polarization on a given sample. Moreover, **o** also represents the Raman shift evolution for three main points in the complex domain structure representing *a*-domain, *a–c*-domain wall and *b*-domain, which are inscribed as **1→1′**, **2→2′** and **3→3′** in **h**–**n**, respectively. The error bars show the s.d. of the Raman shift evolution for three main points in the domain structure for each angle of light polarization on a given sample.

Figure 4a–g shows sequential depth scan Raman images obtained for various *θ* angles. The line **D**→**E** in Fig. 4a–g serves as a guide to the eye to follow the domain motion. Clearly, ferroelectric domains move along the *x*-axis for both the *b*-domains (green) and the *c*-domains (blue). When the polarized light reaches *θ*=90°, the Raman spectrum of the *c*-domain evolves towards *b*-domain (Fig. 4a–g.), accounting for a structural change (more information about the Raman spectra denoted as **D** and **E** in Fig. 4a–g are shown in the Supplementary Fig. 3). The *a*-domain (red), located on top surface of the sample, appears as unchanged. The local change of domain configuration seems to imply the displacement of the *b*-domain (green) to the detriment of the *c*-domain (blue). This observation supports our previous hypothesis of charged *b*-domains: these charges are highly sensitive to electric field and associated to the motion of domain walls.

However, the domain wall motion observed here implies a modification of tetragonality and polarization, at least at local scale. The Raman shift is an indicator of the crystal stress and correlated with both tetragonality and polarization^40^,^41^. Indeed, a modification of the chemical environment (atomic displacement, local stress...) changes the force constants of chemical bonds and thus modify the phonon frequency of some Raman modes (Raman shift). By analogy with the visible spectrum and compared with reference, a frequency increase of the Raman peak frequency is called ‘blueshift’ (higher energy of phonon), while a frequency decrease is called ‘redshift’. Raman shifts reveal chemical bond variations in BO_6_ octahedra, associated with the crystalline stress: We used confocal Raman mapping to understand this complex domain structure. The A_1_(TO_2_) Raman active phonon is a symmetrical mode, which is detected as relatively strong scattering signals in BTO because of a near-perfect equilateral octahedral symmetry. Figure 4h–n displays depth scan Raman shift images related to the A_1_(TO_2_) Raman mode, for the different light polarization angles. Raman shifts as large as ~15 cm^−1^ (from 205 to 220 cm^−1^) are determined in the complex domain structure.

The higher Raman shift corresponds to *c*-domains (blue) near the *a–c*-domain wall. This implies a higher tetragonality in this region and as a consequence a higher off centering of the Ti-cation, which means higher polarization. The surprising increase of polarizability could be explained by the presence of local charge accumulation at the 90° domain wall. The higher Raman shift is thus an evidence of the enhancement of the local electric field provided by the head-to-head *a–c*-domain wall. Besides, the local electrical field also competes with the compressive stress. On the contrary, the *b*-domain (point **3** in Fig. 4h) represents the redshift regions as a consequence of the subdomain structure, in which higher domain density reduces the stress and also the tetragonality.

The Raman shift also evolves with the light polarization angle, with a translation of the domain structure, as stated above. The *a–c*-domain wall moves and the region with local charge accumulation moves accordingly, undergoing redshift with the light angle. The redshift seems to be more relevant for *θ*>45° and the most relevant redshift occurs in the *a*-domain, indicating stress relief, or to some extent a structural modification of the crystal lattice. As a whole, the domain structure promotes a polarization reduction by the effect of the polarized light that originated the domain motion. It must be taken into account that the light is always in-plane polarized: the light polarization is parallel to the *a*-domain polarization at *θ*=0° but perpendicular at *θ*=90°. The redshift variations denote a coupling between the domain orientation and the coherent light polarization.

Figure 4o summarizes the extent to which the domain position is affected by the light polarization angle. This relative motion is illustrated by using as a reference the line **F→G** in Fig. 4a–g. This analysis shows that the relative motion of the domains is ~2.16±0.09 μm when the light polarization direction changes from 0 to 90°. Accordingly, the relative domain displacement presents two different regimes as a function of the light polarization angle. From 45–90°, the relative motion approximately triplicates the one observed between 0–45°, Fig. 4d–g. In addition, there is a progressive change of the *c*-domain nature that reaches the top of the single crystal surface, which becomes *b*-domain in nature. The previous experiments are attempted in two ways: the first one, the light polarization angle increasing in steps from 0°→90° and thereafter from 90°→0°, and the second one the light polarization angle randomly switching from the selected steps. In all cases the domain motion is reversible and remains under the error bars of Fig. 4o. In addition, the experiments are performed on different days and different regions along the complex domain structure, reaching similar results that confirm the reproducibility.

Figure 4o also represents the Raman shift for three main points in the complex structure, representing *a*-domain, *c*-domain and *b*-domain (points **1**, **2** and **3** in Fig. 4h,o, respectively). These three representative points undergo a redshift for the A_1_(TO_2_) Raman mode. When the polarization light rotates between 45 and 90°, the redshift is three times higher than in the 0°–45° range. This fact clearly shows a correlation between the domain wall relative motion and the structural changes. The point **2** (*c*-domain, near of *a–c*-domain wall, out-of-plane polarization) presents a higher stress and higher tetragonality, less sensitive to the light polarization angle. The presence of a local electric field or stress concentration effects seem to be more relevant than the out-of-plane polarization in the *c*-domain region close to *a–c*-domain wall.

## Discussion

**Figure 5:**
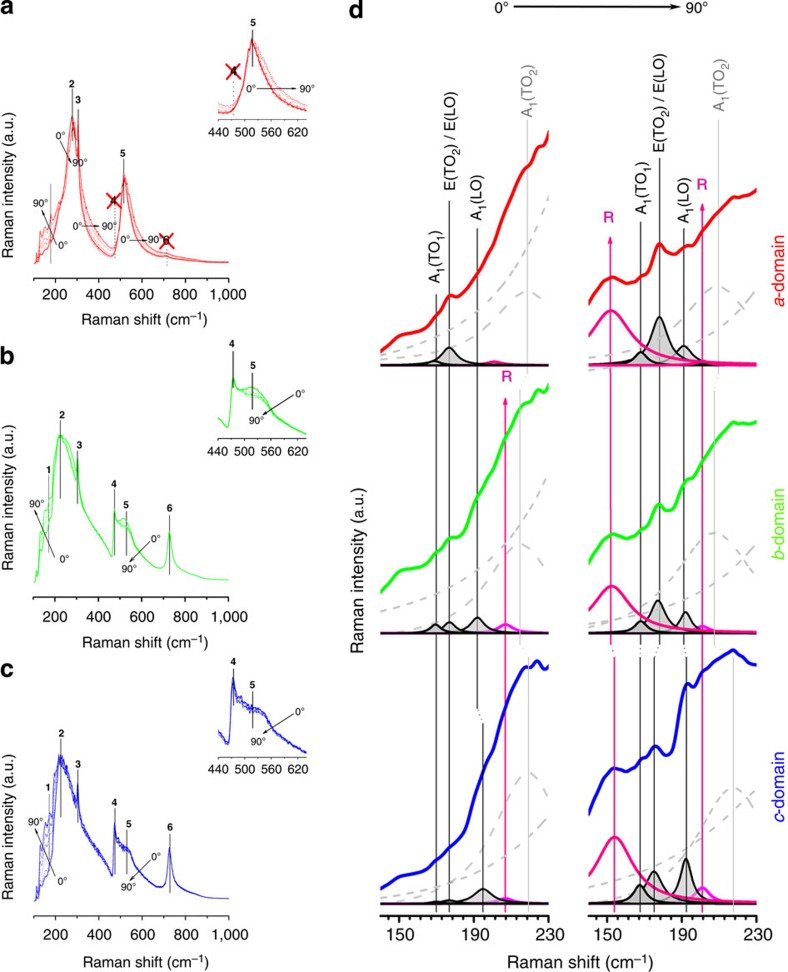
Effect of the light polarization angle in the Raman modes of BTO: The Raman spectra evolution as a function of the light polarization angle representing *a*-domain (**a**), *b*-domain (**b**) and *c*-domain (**c**); which are strategically collected in **1→1′**, **2→2′** and **3→3′** in Fig. 4h–n, respectively. The inserts show magnified Raman spectra, ascribed to the **4** and **5** Raman modes, respectively. (**d**) Comparison of Raman spectra at low wave number region for main domain structures at *θ*=0° (left) and *θ*=90° (right). The light polarization angle value of each image is indicated on the top of **d**. (The numbers next to the Raman peaks represent assignments). The rhombohedral type Raman modes associated to the BTO phase are signalled as R in the Raman spectra of the **d**.

The occurrence of domain motion induced by polarized light is related to the energy provided by photons. Such switching is not totally surprising since the energy provided by the laser is huge when compared, for example, with the tetragonal to cubic phase transition enthalpy (1 J g^−1^, that is, ≈6 J cm^−3^ or 6 pJ μm^−3^). Since the angular dependence of polarized light is observed, the origin of such phenomena is clearly correlated to the interaction between the electric field of polarized light and charges or electrical dipoles, such as the bound charge associated to the head-to-head 90° domain wall or the polarization vector of tetragonal BTO. To analyze such coupling, we studied the evolution of the Raman spectra of surface *a*-domain, *b*-domain and *c*-domain as a function of the light polarization angle (Fig. 5). The Raman spectra are modified with the increasing polarization angle in two main aspects: first, higher intensity in the low wave number region (<200 cm^−1^) and appearance of new Raman modes (Fig. 5d), and second larger asymmetry for the *a*-domains spectra, as shown in the insert of the Fig. 5a. A detailed explanation of these features can be followed below.

First, at low wave number, tetragonal BTO presents Raman modes, assigned to A_1_(TO) at 170 cm^−1^, E(TO_2_)/E(LO) at 180 cm^−1^ and A_1_(LO) at 185 cm^−1^ (refs 42, 43, 44, 45, 46, 47, 48), involving vibrations of Ti atoms against the oxygen octahedra^49^. Raman modes assigned to rhombohedral structure, at 161 and 199 cm^−1^, are normally only observed for temperatures <193 K^27^. Splitting of Raman modes in low wave numbers is characteristic of rhombohedral single crystal, with the appearance of 171 and 187 cm^−1^ Raman modes^24^. In our BTO Raman spectra, differences are detected in comparison with literature (Fig. 5d). The tetragonal structure is clearly stated, with slight differences in Raman shift for the main Raman modes, attributed to the experimental set-up. In addition, forbidden rhombohedral Raman modes appear (Fig. 5d). For *θ*=0°, the *c*-domain Raman mode assigned to E(TO_2_)/E(LO) at 178 cm^−1^ is almost missing, which are sensible to the polarization direction. Thus, the lower presence in *c*-domain denotes an out-of-plane polarization^20^. The appearance of rhombohedral Raman modes for *θ*=0° is incipient because of the Raman mode splitting at 192 cm^−1^ (associated with blueshift of A_1_(LO) for tetragonal symmetry) and 201 cm^−1^. This effect is less clear in *b*-domain and absent in *a*-domain.

The *θ*=90° Raman spectrum shows a different scenario in which Raman modes ascribed to rhombohedral structure gained intensity. Raman modes are evidenced at 154, 193 and 199 cm^−1^ (Fig. 5d). The *c*-domain shows Raman modes with the rhombohedrally distorted symmetry and the relevant presence of E(TO_2_)/E(LO). This means that the *c*-domain changes from out-of-plane to in-plane polarization as a consequence of the rotation of the polarization direction. However, there is a coexistence between <001>_pc_ and <111>_pc_ polarization directions, probably associated with nanometer-size regions^25^. In addition, the redshift of E(TO_2_)/E(LO) Raman mode for the *c*-domain, in comparison with *a*-domain, indicates a structural change as a consequence of the same polarization rotation. It is worth remarking that this situation occurs in the *c*-domain and is related to the application of light polarized perpendicularly to the polarization direction. Thus, the rotation of the polarization from <001>_pc_ to <111>_pc_ is a direct effect of the energy transfer from the polarized light to the crystalline structure. The stress reduction is, however, limited because the out-of-plane polarization is in compressive stress and the in-plane switching is mechanically clamped. The *b*-domain produces always an intermediate response between *a*-domain and *c*-domain response. The *b*-domain Raman redshift denotes a stress reduction as a consequence of the higher domain density of the subdomain structure.

For *a*-domain, the increase of asymmetry observed in Raman mode at 560 cm^−1^ (insert of Fig. 5a), could be analyzed under the same feature. In this case, the <001>_pc_ polarization direction is parallel to the light polarization at *θ*=0°. The change of the light polarization angle also induces the change of ferroelectric polarization to <111>_pc_ direction but still remains in-plane. In all domains, the polarization is enhanced when the polarized light overpasses 45°, because for such angle the ferroelastic restrictions are reduced. Moreover, the asymmetric behaviour of the Raman mode correlates with the appearance of nanometer-size regions in which the polarization rotates^25^. For the *a*-domain, the in-plane polarization rotation enhances the asymmetry accordingly to the light polarization angle. That is, the Raman mode at 560 cm^−1^ originally attributed to the same A_1_(TO_3_) Raman mode is a feature of the appearance of polarization rotation towards the <111>_pc_ direction. The distorted structure possesses higher bonding constant force for such Raman mode and higher blueshift as well. From a structural point of view, this distorted structure appears as a local symmetry, allowing the rotation of the polarization vector during the domain wall motion. The concomitant observation of Raman modes characteristic of BTO rhombohedral polymorph allows thinking that this local structure is probably of rhombohedral symmetry (although monoclinic structure is not disregarded), thus with polarization along the <111>_pc_ direction. This agrees well with the recently reported observation by transmission electron microscopy of the local structure of rhombohedral symmetry in the orthorhombic and tetragonal polymorphs of BTO^25^.

In summary, the ferroelectric domain wall motion induced by a polarized coherent light source at room temperature has been successfully demonstrated in BTO single crystal. The differences between ferroelastic and ferroelectric directions yield a complex domain structure with characteristic 90° domain walls forming <45° from the top surface. On this 90° domain wall, the *a–c*-domain wall is under compressive stress. This stress promotes a complex domain structure, in which *a*-domain, *c*-domain and *a–c*-subdomain regions (named *b*-domain in this work) increase the domain density to minimize stress. We observed different BO_6_ octahedra by the appearance of forbidden phonons in the Raman spectra as a signature of the occurrence of nanometer-sized local structures with <111>_pc_ polarization direction. The <001>_pc_ polarization rotation towards <111>_pc_ occurs through the coupling of the polarized light with the crystalline structure. Thus the polarized light transfers its energy to the crystalline structure to effectively reduce the local mechanical stress. There is a clear correlation between the domain wall relative motion and the light polarization angle. The relaxation of the crystal structure is evidenced by the Raman modes redshift for the different domain regions. The domain wall motion is constricted by local compression stresses and in the case of out-of-plane polarization the relaxation is limited, while the in-plane is more favoured.

To conclude, the implication derived from this study is that we can surprisingly modulate the motion of the ferroelectric domain walls by varying the polarization angle of a coherent light source. The BTO single crystal is relatively simple in domain structure when compared with ferroelectric ceramics and in particular with morphotrophic phase boundary piezoelectric, as PZT ceramics, in which coexistence of different symmetries are on the base of superior piezoelectric properties. It is worth pointing out that this stimulant behaviour observed here could have potential technological applications leading to a non-contact read-out method in FeRAM devices or remote control of piezoelectric actuators. These findings undoubtedly open questions to be solved by the scientific community as well new application possibilities for ferroelectric materials.

## Methods

### Sample preparation

The BTO single crystal used in this study, hereafter referred as ‘BTO’, was produced by top-seeded solution growth and provided by PIKEM Ltd (UK). The 5 × 5 × 1 mm BTO crystal was grown with (100) orientation or *a*-plane. The single-crystal was polished using 1 μm diamond paste, then cleaned with acetone and ethanol before characterization. No further thermal and/or chemical etching was used to reveal the domain structure, thus avoiding topographic artifacts due nanoroughness. The sample was maintained at *T*>25 °C during the AFM and CRM using a temperature controller. In addition, the reader can find more information about the full domain structure of the BTO single crystal in Supplementary Fig. 4. A basic identification of the structure (tetragonal symmetry) and of the orientations of the single crystal (*c*-plane and *a*-plane) was performed using X-Ray Diffraction (X'Pert PRO Theta/2theta of Panalytical, Cu K_α_ radiation). The lattice parameters were refined by a global simulation of the full diagram (pattern matching, FullProf programme). The orientation degree was determined using the intensity ratio of the two characteristic diffraction peaks I(002)/I(200).

### AFM and CRM domain mapping

The experiments were performed using a CRM coupled with AFM (Witec alpha-300R). The sample was imaged in AFM AC mode using ArrowFM cantilevers (Nanoworld, Germany) with a resonance frequency in the range of 70–90 kHz and damping of *r*=50%, recording both topography and phase images simultaneously. Raman spectra were obtained using a frequency-doubled Nd-YAG laser operating at 532 nm and a × 100 objective lens (numerical aperture=0.9). The incident laser power was 40 mW. The optical diffraction resolution of the confocal microscope was limited to about ~250 nm laterally and ~500 nm vertically. Raman spectral resolution of the system was down to 0.02 cm^−1^. The microscopy sample was mounted on a piezo-driven scan platform having 4 nm lateral and 0.5 mm vertical positional accuracy. The piezoelectric scanning table allows steps of 3 nm (0.3 nm in the vertical direction), giving a very high spatial resolution for both the AFM and the CRM. The microscope base was also fitted with an active vibration isolation system, active 0.7–1000 Hz. Collected spectra were analyzed by using Witec Control Plus Software.

### Angular dependence

The sample was mounted under the microscope objective with the domains walls aligned parallel to the *y*-axis of the piezo-driven scan table and perpendicular to the *x*-axis. The light incidence was normal to the sample surface, the polarization direction of light was parallel to the surface, and the scan direction, denoted as *x*-axis, was maintained perpendicular to the *a–c*-domain wall. The *in situ* domain switching was monitored taking subsequent depth scans at different polarization angles of the same area, using as position reference a significant point of the sample surface visible with the optical microscope. Depth scan Raman images had 10 μm of width, 4 μm of height, 100 × 40 spectra of 0.2 s of integration time at 40 mW of laser power. Thus, the Raman image consisting of 100 × 40 pixels (4,000 spectra) required ~14 min for acquisition of the whole cross-section. In addition, to avoid the photoconductivity effects present in BTO, in our experiments a polarized light with a wavelength (at *λ*=532 nm) outside the two absorption maxima^18^,^19^ is chosen and the open circuit is selected. The angle between the polarization of light and the depth scan direction by using Δ*θ*=15° between *θ*=0° and *θ*=90° was rotated. Experiments were attempted in two ways: first, the light polarization angle varying in steps from 0°→90° and thereafter from 90°→0°, and second the light polarization angle randomly switching from selected steps. Collected spectra were analyzed by using Witec Control Plus Software.

## Author contributions

F.R.-M. designed and performed the experiments, analyzed the results and wrote the manuscript. A.D.C. carried out AFM and Raman Image experiments. P.M. assisted in experiments and contributed to data analysis and the writing of the manuscript. J.F.F. initiated and supervised the project and contributed to the writing of the manuscript.

## Additional information

**How to cite this article:** Rubio-Marcos, F. *et al*. Ferroelectric domain wall motion induced by polarized light. *Nat. Commun*. 6:6594 doi: 10.1038/ncomms7594 (2015).

## Supplementary Material

Supplementary Information Supplementary Figures 1-4, Supplementary Tables 1-2 and Supplementary References
