# Supplementary material for: Ferroelectric domain wall motion induced by polarized light
Source: Nat Commun. 2015 Mar 17;6:6594. doi: 10.1038/ncomms7594 (PMC4382678; doi:10.1038/ncomms7594)
Supplement: Supplementary Information — Supplementary Figures 1-4, Supplementary Tables 1-2 and Supplementary References [file ncomms7594-s1.pdf]

Supplementary Figures

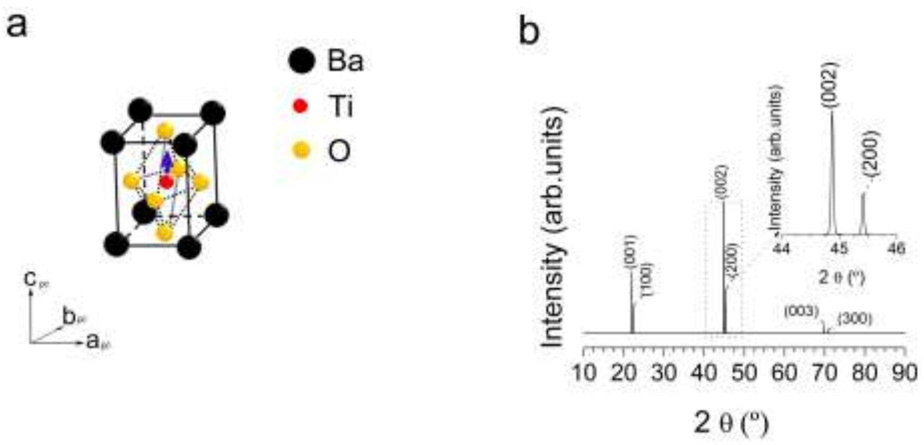

Supplementary Figure 1 | Structural Characterization of the BTO single crystal by XRD: The panel **(a)** shows the unit cell of *P*4*mm* $BaTiO_3$. In tetragonal *P*4*mm* $BaTiO_3$ (BTO), ferroelectricity is mainly due to a relative displacement along the *c*-axis of titanium from its centrosymmetric position in the unit cell and in the oxygen octahedra. In correlation with titanium shift, barium ions are also displaced along the fourfold axis of the tetragonal cell. A permanent electric dipole, or ferroelectric polarization, is thus created (blue arrow in panel **a**). The deviation of the c/a ratio from unity is used as an indication of the presence of the ferroelectric phase. **(b)** XRD pattern of the BTO single crystal. A basic identification of the structure and of the crystalline orientation of the single crystal studied was thus performed by using X-Ray Diffraction. The **inset** of the **Fig. b** shows a detail of the XRD diffraction pattern in the 2Θ range 44° to 46°, corresponding to (002) and (200) peaks of the tetragonal symmetry. The absence of most of the diffraction peaks observed for polycrystalline samples (powder), especially the most intense (101)/(110) double peak at 31.5-31.6°, confirm the single-crystal nature of the sample. Only two families of peaks, (00l) and (h00), are detected, indicating that: the sample is poly-domain and present two different orientations. Indeed, a pure $<001>_{pc}$-oriented BTO single crystal should present only one family of (00l) peaks. Furthermore, the splitting of the $(00l)_{pc}$ peaks into (00l) and (100) confirms the tetragonal symmetry of the sample (see panel b), with lattice parameters a = 3.9899 Å and c = 4.0340 Å (see **Supplementary Table 1**), in agreement with reported values.[1] The relative intensity of (100) and (00l) peaks are reversed compared to powder data. Thus, the (00l) orientation, or *c*-plane, is the dominant one. To sum up, the samples presents a tetragonal symmetry and two different crystallographic orientations, (00l) or *c*-plane and (100) or *a*-plane.

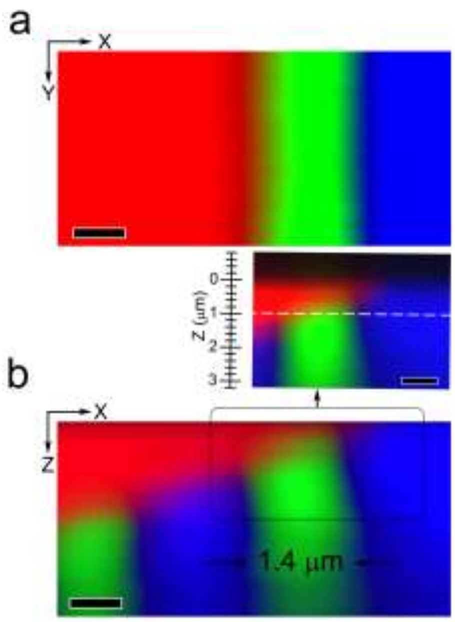

Supplementary Figure 2 | Characterization of the BTO single crystal through Confocal Raman spectroscopy: Magnified Raman image shows the domain distribution at the surface by colour code **(a)** and in the depth scan (i.e. cross section) **(b).** The series of Raman images **(a-b)** display a domain structure composed of *a*-domain (red domain), *c*-domains (blue domains) and *b*-domain (green domains) that appear in the *a*-*c*-domain wall within the ($\{101\}_{pc}$) plane that slopes ca. 20º to the top surface, such as shown in panel **b**. The main Raman spectra of BTO Raman image associated with different colours can be observed in the Fig. 2d of the main manuscript. We found more than one *b*-domain (shown in green) that hindered only the *a*-*c*-domain wall. There is a set of *b*-domains parallel between them that grow in the c-domain region. The Raman spectrum of the *b*-domain is a clear combination between *a*-domain and *c-d*omain Raman spectra, see Fig. 2d of the main manuscript. The *b*-domains are characterized by exhibiting an average width of ca. 1.4 µm (see panel b). The insert localized in the top of the panel b shows three main regions in the complex structure representing *a*-domain (red domain), *c*-domain (blue domains), and *b*-domain (green domains). The white dash line shows the positions where the XY Raman image is performed and correspond with a focal plane at Z=1 µm. Scale bar, 1 µm**.**

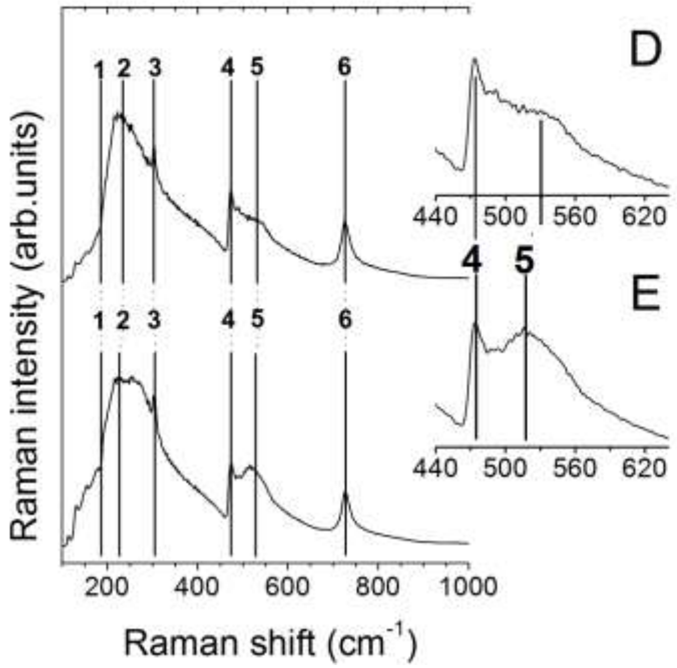

Supplementary Figure 3 | Schematic of the BTO complex domain structure: The Raman spectra taken in two strategic points select along the line direction that we have denoted as **D,** and **E** in the set of **Figs. 4a-g** of the main manuscript. The Raman spectrum localized at the top of the **Supplementary Fig. 3** corresponding to *c-domains,* which is depicted as a blue region in the **Fig. 4a** of the main manuscript. When the angular rotation of polarized light is increased until 90º, the Raman spectrum evolves toward a ferroelectric *a-c-domain* state (green region in the Fig. 4g of the main manuscript).

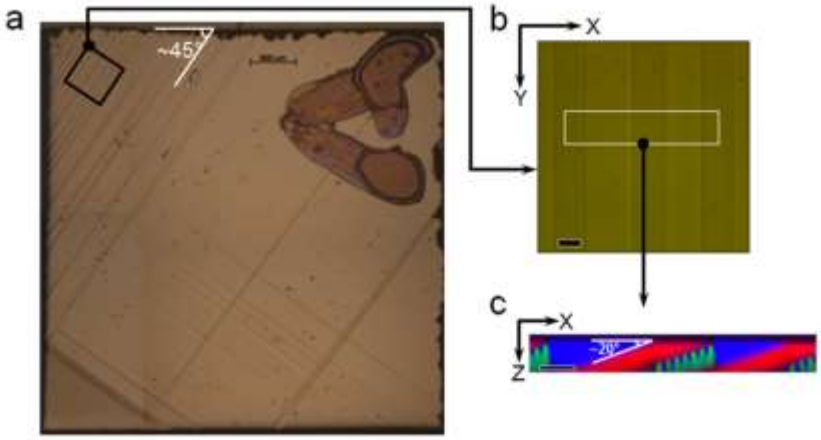

Supplementary Figure 4 | The panel **(a)** shows an optical microscopy image of the studied BTO single crystal, in which a complex domain structure is observed, showing the presence of lamellar twinning. The domain structure is mainly composed 90° domain walls forming striped regions, aligned at 45 º of sample's edges. The width of the domains is not periodic and varied from adjacent domains. (The pencil mark in the upper right corner is just a sample reference). **(b)** Magnified optical image of the BTO single crystal where it is shown a detail of the domain structure in the marked black box on the panel (a), which correspond with the area of main body ´s AFM analysis. Scale bar, 20 µm. For the mapping of the domain structure of the BTO single crystal through Confocal Raman Microscopy, the sample was mounted under the microscope objective with the domain walls aligned perpendicularly to the Raman laser. Therefore the BTO single crystal was rotated 45º between pictures (a) and (b) in order to align the domain walls with the Y-axis of the reference system used for Raman experiments. The white rectangle of panel b shows the positions where the *XY* Raman image and *XZ* Raman depth scan image are performed. **(c)** *XZ* Raman depth scan image. From the panel **c**, it can be observed the 90º-domain walls have 20º slopes to the surface. Scale bar, 20 µm.

Supplementary Tables

Supplementary Table 1. Unit-cell parameters of $BaTiO_3$ single crystal, in the tetragonal *P*4*mm* structure, at room temperature. Structural refinement results of the XRD pattern shown in **Supplementary Figure 1**, which recorded at room temperature.

	**a** [Å]	**b** [Å]	**c** [Å]	***c*/a**	**Volume** [Å^3]	***z-orientation*** (%)
**BTO single crystal**	3.99117 ± 0.00004	3.99117 ± 0.00004	4.03632 ± 0.00006	1.01131	64.296 ± 0.001	74.4 ± 0.2

Supplementary Table 2. Raman modes and their mode symmetry assignments in tetragonal $BaTiO_3$ single crystal**. Table** summarizes both symmetry and nature (first and second order), of the Raman modes of the $BaTiO_3$ phase. According to the nuclear site group analysis, Raman active phonons of the tetragonal *P*4*mm* (C_{4v}^1) crystal symmetry are represented by $3A_1 + B_1 + 4E$. Long-range electrostatic forces induce the splitting of transverse and longitudinal phonons, which results in split Raman active phonons represented by $3[A_1 (TO) + A_1 (LO)] + B_1 + 4[E (TO) + E (LO)]$.

**Raman Shift** (cm^{-1})	**Symmetry**	**Abbreviated** number	**Reference**
36	E (TO)	**1**	2-6
170	A_1 (TO)		3-8
180	E (TO_2), E (LO)		2-6
185	A_1 (LO)		3-8
210-270	A_1 (TO_2)	**2**	3-8
305	E (TO_3 + LO_2)	**3**	2-6
305	B_1		2-6
463	E (LO_3)	**4**	2-6
475	A_1 (LO_2)		3-8
486	E (TO_4)		2-6
518	E (TO_5)	**5**	2-6
520	A_1 (TO_3)		3-8
715	E (LO_4)	**6**	2-6
720	A_1 (LO_3)		3-8

Supplementary References

1. Ghosh, D., Sakata, A.; Carter, J., Thomas, P.A., Han, H., Nino, J.C., & Jones, J.L. Domain Wall Displacement is the Origin of Superior Permittivity and Piezoelectricity in $BaTiO_3$ at Intermediate Grain Sizes. *Adv. Funct. Mater.* **24**, 885–896 (2014).
2. DiDomenico, M., Wemple, Jr., S. H., Porto, S. P. S., & Bauman, R. P. Raman Spectrum of Single-Domain $BaTiO_3$. *Phys. Rev.* **174**, 522-530 (1968).
3. Venkateswaran, Uma D., Naik, Vaman M., & Naik, Ratna. High-pressure Raman studies of polycrystalline $BaTiO_3$. *Phys. Rev.B* **58**, 14256 (1998).
4. Dobal, P. S., & Katiyar. R. S. Studies on ferroelectric perovskites and Bi-layered compounds using micro-Raman spectroscopy. *J. Raman Spectrosc.* **33**, 405-423 (2002)
5. Shiratori, Y., Pithan, C., Dornseiffer. J., & Waser. R. Raman scattering studies on nanocrystalline $BaTiO_3$ Part I – isolated particles and aggregates. *J. Raman Spectrosc.* **38,** 1288-1299 (2007)
6. Shiratori, Y., Pithan, C., Dornseiffer. J., & Waser. R. Raman scattering studies on nanocrystalline $BaTiO_3$ Part II – consolidated polycrystalline ceramics *J. Raman Spectrosc.* **38,** 1300-1306 (2007)
7. Pinczuk, A., Taylor, W. T., Burstein, E., & I. Lefkowitz, I. The Raman Spectrum of $BaTiO_3$. *Solid State Commun***.** **5**, 429-433 (1967).
8. Burns, Gerald., & Scott, Bruce. A. Raman Scattering in the Ferroelectric System $Pb_{1-x}Ba_xTiO_3$. *Solid State Commun.* **9**, 813-817 (1971).
